# The Effect of Maternal Relaxation Training on Reactivity of Non-Stress Test, Basal Fetal Heart Rate, and Number of Fetal Heart Accelerations: A Randomized Controlled Trial

**Published:** 2015-01

**Authors:** Marzieh Akbarzade, Bahare Rafiee, Nasrin Asadi, Najaf Zare

**Affiliations:** 1 Community Based Psychiatric Care Research Center, Department of Midwifery, School of Nursing and Midwifery, Shiraz University of Medical Sciences, Shiraz, Iran;; 2 Department of Midwifery, School of Nursing and Midwifery, Shiraz University of Medical Sciences, Shiraz, Iran;; 3 Department of Obstetrics and Gynecology, School of Medicine, Shiraz University of Medical Sciences, Shiraz, Iran;; 4 Department of Biostatistics, School of Medicine, Infertility Research Center, Shiraz University of Medical Sciences, Shiraz, Iran

**Keywords:** Accelerations, Fetal Heart, Heart Function Test, Relaxation

## Abstract

**Background:** Relaxation-training, as an anxiety-reducer intervention, plays an important role in fetal health. The present study aimed to analyze the effect of maternal relaxation on stress test (NST), basal fetal heart rate, and number of fetal heart accelerations.

**Methods:** In this randomized controlled trial, 84 pregnant women were randomly divided into two groups of teaching relaxation and control groups in 2012. In the intervention group, 60-90 minute classes were held every week lasting for 4 weeks. Besides, home practice charts were given to the mothers and researchers controlled the home practices by phone calls every week. The control group received routine prenatal care. In the 4^th^ week, NST was performed in the intervention group 30 minutes before and after the 4^th^ session. In the control group, NST was done in the 4^th^ week. The quantitative variables in the two groups were compared through ANOVA and Chi-square test.

**Results: **The results of paired t-test showed that relaxation could improve the NST results (P=0.01). Mean and standard deviation of basal fetal heart rate was 138.95±8.18 before the intervention and 133.07±6.9 after the intervention. Paired t-test also showed that relaxation reduced the basal fetal heart rate (P=0.001). Mean and standard deviation of the number of fetal heart accelerations was 1.5±0.8 before the intervention and 2.2±0.9 after it. The results of paired t-test also showed that relaxation increased the number of fetal heart accelerations (P=0.001).

**Conclusions: **Relaxation could improve the NST results, reduce the basal fetal heart rate, and increase the number of fetal heart accelerations. Therefore, relaxation is recommended during pregnancy.

**Trial Registration Number**: IRCT2012072810418N1

## Introduction

Relaxation is known as an anxiety-reducer intervention that has great effects on both mother and fetus. Different ways of relaxation can significantly reduce the state and trait anxiety in nuliparous women.^1^ A study conducted in Iran showed that relaxation could significantly reduce the anxiety disorders.^2^ The importance of the anxiety accounts for the fact that the children whose mothers were anxious during pregnancy will have low academic education,^3^ increased behavioral-emotional problems in adulthood,^4^ decreased mental function and evolution of language,^5^ and increased incidence of psychopathology in adulthood.^6^ Anxiety also has a devastating impact on infant’s motor development, cognitive ability, and the ability to match the emotional^7^ and neonatal behavior^8^ during pregnancy. Researchers have found that anxiety at 32 weeks of pregnancy can predict emotional problems, including hyperactivity behavior,^9^ autism, and disorders of reading and writing^10^ in adulthood. Researchers have shown that maternal mental- psychological condition influences the fetal heart rate patterns. Thus, the fetus responds to maternal mental changes that indicates maturity and evolution of fetal autonomic nervous systems. Recently, fetal response to maternal anxiety has made the researchers offer a hypothesis suggesting that the fetus inside the mother’s body is very sensitive and responsive to the environment. Therefore, the changes in the maternal sympathetic system due to anxiety, such as increased heart rate and changes in diastolic blood pressure, cause signal transmission to the fetus.^11^ Some researchers found that depressed mothers had increased basal fetal heart rate and 3.5 times delay in returning to the baseline after vibroacoustic stimulation. In these women, the fetal heart rate pattern was more biphasic (rising and falling quickly) and the average of the maximum distance with the rising phase was significantly shorter than that of the control group.^12^

Other researchers found an increase in the fetal heart rate of the mothers with high anxiety levels during pregnancy.^13^ Pregnancy is a stressful event which may increase morbidity and mortality. Studies have shown depression, anxiety, and other mental health conditions in pregnancy as the risk factors for fetal loss, preterm birth, and low birth weight.^14^ Thus, the interventions which reduce the maternal anxiety will have an important role in fetal and maternal health. Non-pharmacologic interventions are based on mind- body medicine. The main focus of these interventions is on the interaction between mind and body and its impact on health. Interventions in mind-body medicine have asserted the use of skills which increase the capacity of the mind to influence the body functions. These interventions include hypnosis, visual imagery, yoga, and relaxation.^15^ There are various relaxation techniques, some of which, such as progressive muscle relaxation, are used to reduce muscle tension. In other techniques, such as Benson, the main purpose of the exercise is reducing the sympathetic anxiety symptoms.^16^ Parental training classes provide women with opportunities to talk about their fears and concerns and learn how to reduce their anxiety.^17^ Benson defined four major fundamental elements in the relaxation as follows: 1. Quiet place for relaxation, 2. A convenient position, 3. Something to focus on, and 4. Trying to maintain attention and concentration.^16^ Relaxation mediates anxiety reduction and can have an important role in physiological functions. Probably, the relaxation mechanism is to regulate the hypothalamus action and decrease the sympathetic nervous activity. Relaxation response is a physiological phenomenon which is activated by parasympathetic nervous system and reduces the anxiety by facilitating the release of endorphins. Following the relaxation of muscles and sympathetic to parasympathetic dominance, one can feel comfortable. In fact, performance of physical activities causes the patients to be distracted from other issues, pay attention to be directed toward the physical activity, spend more time in calmness, and be less irritable and anxious in future.^18^^,^^19^ Other researchers showed that maternal diastolic blood pressure declined due to relaxation. Decreased sympathetic system activity and heart rate was also evident. Fetal heart beat to beat variability was increased, as well.^20^ Another study revealed that playing music for pregnant women reduced the sympathetic activity, which is a major cause of increased cardiac arrhythmia, during pregnancy, thus improving the heart function.^21^ Another researcher studied the effects of relaxation on the heart function in patients with heart disease. The results of that study showed that after training the patients on relaxation, plasma norepinephrine levels and stroke volume decreased, while end diastolic volume increased.^22^ This means that relaxation reduces the stress hormones and increases the heart relaxation time. In another study, it was revealed that the average heart rate reduced to 4 beats per minute due to relaxation.^23^ Considering blood pressure changes, other studies showed that relaxation led to 10.6 and 4.8 mmHg reduction in systolic blood pressure and diastolic blood pressure, respectively in 14 patients with hypertension.^24^ Therefore, the present study aims to determine the effect of Benson’s relaxation reaction training on non-stress test, baseline fetal heart rate, and number of fetal heart accelerations.

## Materials and Methods

The present randomized clinical trial was approved by the Ethics Committee of Shiraz University of Medical Sciences. We aimed to assess the effect of relaxation training on non-stress test parameters. The calculation of sample size was made by the following formula and   involved the variance of 0.92, the minimum difference mean 0.6 , type I error equal to 5% , and the power equal to 80%; the significance level was considered p< 0.05 (σ1=σ2, d=2,  α=0.05, β=0.1). The sample size was calculated to be 72 participants; the potential loss of 84 was considered. Sampling was   purposive and mothers who had the inclusion criteria were enrolled in the study.

$$n=\frac{2\left({\text{z}}_{1-\frac{\alpha }{2}}+{\text{z}}_{1-\beta }\right)\delta^{2}}{{\text{d}}^{2}}$$

**Figure 1:**
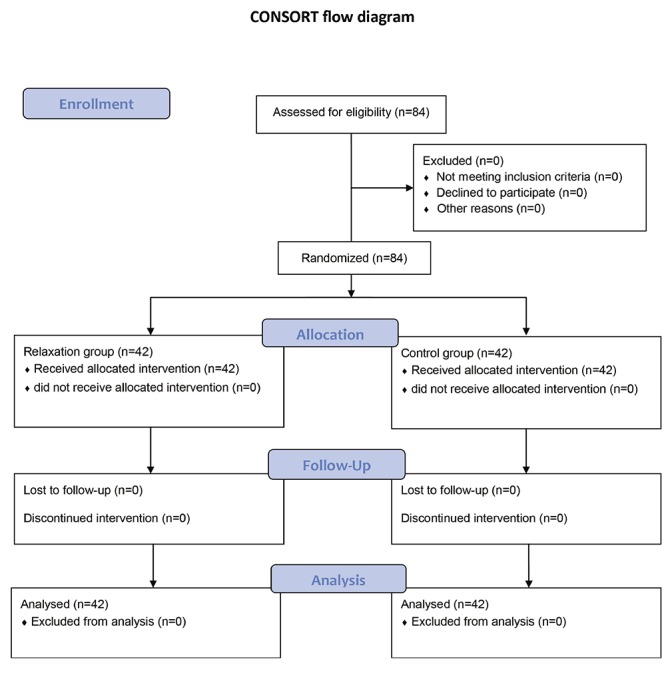
CONSORT flow diagram of participants

84 pregnant women were enrolled randomly an84 pregnant women were enrolled randomly and divided into two groups of relaxation and control (Figure 1). The randomization was done in the draw; the groups were selected so that the first prize in each center was randomly and slowly drawn in the relaxation intervention group and the second person in the control group, and sampling continued until the number of samples in groups was complete. This study could not be blinded because of the interaction between the pregnant women for relaxation and other mothers. The inclusion criteria of the study were 1. Being Primipara, age between 18 and 35 years old, and being cephalic singleton at the gestational age of 28-34 weeks, 2. Not having anatomical and psychological disorders, such as psychosis, schizophrenia, and uterine and pelvic abnormalities, 3. Not having experienced threatening factors, such as hypertension, decreased fetal movement, intrauterine growth restriction, rupture of membranes more than 12 hours, and history of infertility, from the beginning of pregnancy until the 28th week of gestation, 4. Having a negative history of chronic diseases, including heart disease, pulmonary hypertension, and diabetes, 5. Having at least middle school degree, 6. Being able to regularly attend the classes, 6. Receiving care during pregnancy, and 7. Having no history of drug abuse or smoking. At first, the women were divided into a control and a relaxation training group based on simple randomization.

Relaxation training interventions were held in three groups of 14 members (one session a week each lasting for 60-90 minutes). Relaxation topics included: First session-Introducing the concept of anxiety, symptoms of anxiety, life and pregnancy stress recourses, ways to manage stress, and introducing the relaxation concept and its logic to be applied in pregnancy and life. Second session: Introducing an ideal environment for relaxation and introduction of muscle relaxation techniques. Third session- Familiarity with breathing exercises. Fourth session- Overview of a complete relaxation. In the relaxation group, the classes were held every week for 4 weeks. Besides, Non-Stress Test (NST) was done twice in the intervention groups, once before and once after the 4th session. In the control group, NST was done only once during the 4th week of participation in the study. Gestational age was determined by last menstrual period and, in case of uncertainty, by the first Doppler sonography. NST was done before and after the 4th session because a review of all the previous sessions was performed in the 4th session and we could better determine the impact of the training. Given that a variety of factors have an impact on the fetal heart pattern, the researchers limited the time of conducting the test to the 4th session. In this study, NST was done by an external monitoring device (model Analogic lite- TM, made in Japan) in 20 minutes. This device works with Doppler technology. Acceleration of fetal heart rate is defined as the increase in basal fetal heart which is associated with fetal movements in nearly all the cases. These accelerations are reliable and almost always confirm that the fetus is not academic at that moment. However, the absence of these accelerations is not necessarily a bad sign unless accompanied by other reassuring symptoms. Reduction in such reactions is most commonly seen in association with fetal sleep cycles.^25, 26^

*Statistical Methods*

For measuring the reactivity or lack of reactivity of NST, Chi-square test was used. In addition, the number of accelerations and basal fetal heart rate were measured through Kruskal-Wallis test. Besides, paired t-test was used to compare the variables before and after the intervention. 

## Results

Before the interventions, 53.2% of the NSTs were reactive and 46.8% non-reactive. The statistical test showed no significant differences between the NSTs before the interventions (P=0.96). After a month of intervention, 74.1% of the NSTs were reactive and 28.6% non reactive. Chi-square test (P=0.01) showed that the changes in NST were statistically significant in the relaxation group after the intervention (Table 1). The mean of fetal heart rate (P=0.97) was not statistically significant before the interventions; however, the difference was significant in the relaxation group after the intervention (P=0.001) (Table 2). Before the intervention, the two groups were homogeneous (P=0.9) regarding the number of fetal heart accelerations; nevertheless, relaxation training (P=0.001) increased the number of accelerations (Table 3).

**Table1:** Frequency of reactive and non reactive NST before& after intervention

**NST**		**Control**		**Relaxation**		**Total**	
		**Percent**	**Number**	**Percent**	**Number**	**Percent**	**Number**
Reactive test	Before	54.8%	23	52.4%	22	53.2%	45
	After	64.3	27	78.6%	33	71.4%	60
Nonreactive test	Before	45.2%	19	47.6%	20	46.8%	39
	After	35.7	15	21.4%	9	28.6%	24

Chi-square test (P=0.01)

**Table 2:** Number of fetal heart rate in NST before intervention

**Group**	**Control**	**Relaxation**	**P value**
**FHR**	**Mean±SD**	**Mean±SD**	
Before Intervention	138.6±8.27	138.95±8.18	0.97
After Intervention	138.6±8.27	133.07±6.9	0.001

Paired t-test, P values lower than 0.05 were considered as significant

**Table 3:** Number of fetal heart accelerations before and after interventions

**Group**	**Control**	**Relaxation**	**P value**
**Number of accelerations**	**Mean±SD**	**Mean±SD**	
Before intervention	1.5±0.9	1.5±0.8	0.9
After intervention	1.5±0.9	2.2±0.9	0.001

Paired t-test, P values lower than 0.05 were considered significant.

## Discussion

Before the interventions, 53.2% of the NSTs were reactive and 46.8% non-reactive. The statistical test (P=0.96) showed no significant differences between the NSTs before the interventions. Another researcher studied the effect of music on NST. At first, 47.7% of the total NSTs were non-reactive and 52.6% were reactive. One way ANOVA showed (P=0.07) no significant difference between the two groups before the intervention.^23^ Yet,  a study was conducted on 470 pregnant women in labor. In that study, the last NSTs were compared with the women’s pregnancy outcome. According to the results, 89.4% of the NSTs were reactive and 10.6% were non-reactive.^27^ Differences in percentage and frequency of reactive/non-reactive NSTs are highly dependent on one’s skill and manner of interpretation. To diminish this devastating effect, all the NSTs were interpreted by one gynecologist in this study.

One month after the interventions, 74.1% of the NSTs were reactive and 28.6% were non- reactive. Paired t-test (P=0.01) showed that in the relaxation group, changes in NST were statistically significant before and after the intervention. In the study by Keshavarz, 71% of the NSTs were reactive and 29% were non-reactive^23^ after the intervention, which is similar to this study. It seems that the great effect of relaxation on the cardiovascular function is highly correlated with its anxiety-reduction role. Maternal anxiety can affect the fetal cardiovascular function. Glower et al. showed that maternal cortical level could affect the placental function in anxious women. Stress hormones, such as cortisol, can stimulate the placenta to release corticotrophin in the fetal blood stream which stimulates fetal hypothalamus- pituitary- adrenal axis to produce cortisol. This hormone, as well as others such as epinephrine, can reduce the placental perfusion.^28^ Increase of beat to beat variability and decrease of fetal movements were reported in response to maternal anxiety in another study. Of course, male and female fetuses had the same responses. This study also reported that fetal movements and cardiac responses were more obvious in 36 weeks of gestation compared to 24 weeks.^29^ It seems that anxiety-reduction interventions, like relaxation, massage, and hypnotism therapy have similar effects on maternal-fetal cardiac function. Another researcher found a 17% reduction in oxygen consumption in healthy people during relaxation^30^ so that much oxygen was available for fetal use.

Another study showed that relaxation could result in significant changes in fetal eurobehavior;  there was a decrease in fetal heart rate (FHR), and FHR variability increased.^16^ Studies have also shown that relaxation can reduce hypothalamus-pituitary-adrenal axis, sympathetic system activity, and cortisol level,^31^ thus reducing the blood pressure and heart rate.^32^ On the other hand, studies have shown that increase of beat to beat variability due to relaxation is the most important factor in NST reactivity.^33^ A researcher found significant differences in the level of blood stream before and 30 minutes after massage therapy. They also stated that this technique could affect the physical as well as mental health, leading to relaxation and improving the physiological parameters.^17^ Therefore, it can be stated that relaxation can reduce anxiety and sympathetic system activity. Consequently, levels of hormones, such as norepinephrine and cortisol, are reduced causing a reduction in heart rate, stroke volume, and oxygen consumption, while an increase in the end-diastolic volume and cardiac output. This mechanism is the most probable pattern for explaining why relaxation improved the NST in this study.

The mean of fetal heart rate (P=0.97) was not statistically significant before the interventions; however, the difference was significant in the relaxation group after the intervention (P=0.001). This means that relaxation can decrease the fetal heart rate which is attributed to the fetal sympathetic system activity. Similar results were also obtained in the studies performed on massage therapy. Overall, the effect of maternal anxiety on fetal heart rate is interesting. Talbert et al. reported an increase in basal fetal heart rate in anxious women.^34^ Basal fetal heart rate also increased in the women who were listening to their favorite music.^35^ It seems that the effect of anxiety on fetal heart rate is through the same hormonal mechanism that can reduce the placental perfusion and increase the basal fetal heart rate. According to above cycle, we can expect that relaxation and other similar interventions can reduce the basal fetal heart rate. For example, massage therapy can affect the central nervous system to release central anesthesia, such as B endorphin and encephalin, which can prevent releasing neurotransmitters. As a result, sympathetic system activity is decreased, while parasympathetic system activity is increased and, consequently, heart rate and respiratory rate are reduced. Massage therapy can also relax the muscles, increase the capillary volume, and decrease the blood pressure. A researcher found that massage therapy could reduce the blood pressure from 130.80 to 116.75 in anxious women.^36^

In this study, the mean of fetal heart accelerations was increased, probably due to the anxiety reduction. Up to now, few studies have calculated the number of accelerations. For instance, another researcher studied the effect of maternal anxiety and music therapy on fetal heart accelerations and showed that music therapy increased the number of fetal heart accelerations.^37^

The limitations of the study included: 1. Unwillingness of some participants to cooperate. Of course, the researchers expressed the importance of the topic in the mother and fetus health and involved the women in the discussions in order to motivate more women. 2. Differences in the interpretation of NST. In order to eliminate this limitation, all the NSTs were interpreted by one gynecologist. 

## Conclusion

Relaxation can improve the NST results, reduce the basal fetal heart rate, and increase the number of fetal heart accelerations. Therefore, relaxation is recommended during pregnancy. It is recommended that further studies should be conducted on relaxation in high risk pregnancy 
